# ﻿Geometric Morphometrics sheds light on the systematics affinities of two enigmatic dwarf Neotropical sedges (*Carex*, Cyperaceae)

**DOI:** 10.3897/phytokeys.232.100410

**Published:** 2023-09-21

**Authors:** Ana Morales-Alonso, Tamara Villaverde, Pedro Jiménez-Mejías

**Affiliations:** 1 Área de Botánica, Department of Molecular Biology and Biochemical Engineering, Universidad Pablo de Olavide, ctra de Utrera km 1, Seville 41013, Spain Universidad Pablo de Olavide Seville Spain; 2 Área de Biodiversidad y Conservación, Department of Biology and Geology, Physics and Inorganic Chemistry, Universidad Rey Juan Carlos, Calle Tulipán s/n., Móstoles 28933, Madrid, Spain Universidad Rey Juan Carlos Madrid Spain

**Keywords:** acaulescence, *Carex*, geometric morphometrics, Neotropics, sedges

## Abstract

Geometric morphometrics (GM) is a powerful analytical tool that enables complete quantification of shapes. Its use in Botany has a great potential for complementing plant evolutionary and ecological studies. Taxonomic delimitation in *Carex* has been complicated due to reduction of characters and frequent homoplasy. This problem is more marked in cases where the species exhibit dwarfism. South America is the continent with the least understood *Carex* flora. The systematic relationships of some bizarre-looking groups were not unraveled until molecular phylogenetic studies resolved their relationships. In particular, there are two species only known from their type material whose affinities remain uncertain: *Carexherteri* and *C.hypsipedos*. These two taxa are acaulescent plants that respectively grow in the Uruguayan pampa and Peruvian high-altitude meadows. Recently, both species were ascribed to the *Carexphalaroides* group (subgen. Psyllophorae, sect. Junciformes) due to superficial morphological similarities, such as the androgynous peduncled spikes. However, their character combination is also coincident for its circumscription to sect. Abditispicae species. Nevertheless, in the absence of confirmation from molecular analyses, their placement must be considered preliminary until additional data can be provided. In this work we employ for the first time geometric morphometrics (GM) tools to assess the systematic affinities of two taxonomically problematic sedge species based on fruit shape. We compared utricle morphology of *C.herteri* and *C.hypsipedos* with that of *C.phalaroides* group and species in sect. Abditispicae. To this end we used GM and traditional morphometric approaches. Utricle shape variation along with other morphological features support the exclusion of these two species from the *C.phalaroides* gr. and, at the same time, show clear affinities of *C.herteri* to sect. Abditispicae. *Carexhypsipedos* remains as an *incertae sedis* species. Our work shows the potential utility of GM for the exploration of systematic affinities in sedges and in other graminoids.

## ﻿Introduction

Before the advent of molecular systematics, taxonomic delimitation relied on the evaluation of phenotypic differences. Visible characteristics of organisms have been the basis for classifying the diversity of life within a unifying taxonomic framework. Analytical advances have allowed the implementation and consequent improvement of tools that can be applied to morphology-based studies (e.g. correlation coefficient (Pearson 1895), analysis of variance (Fisher 1935) or principal components analysis (Pearson 1901; Hotelling 1933). However, certain evolutionary phenomena, such as homoplasy, stasis or recent divergence, may have consequences on morphology of the organisms, hampering the distinction of certain taxa based only on morphological characteristics. Accordingly, these types of tests lose their resolving power, making it necessary to search for additional evidence for its distinction.

One of the less explored analytical tools in plant systematics is geometric morphometrics (GM). GM was developed around 1980 (e.g. Kuhl and Giardina 1982), allowing the analysis of structures shapes and their variation. It uses non-quantitative variables through coordinates of landmarks, which collect geometric information on their relative position (Chen et al. 2018). It enables the visualization of multivariate analyses results as a configuration of landmarks from the original spatial configuration of the organism (Adams et al. 2004). This tool uses as a basis the Procrustes analysis of fixed and sliding landmarks, which extracts a consensus configuration (mean) by standardizing effects of rotation, orientation, and scale among specimens. These effects are translated to the origin, scaled to unit-centroid size, and rotated via a generalized least-squares algorithm that enables their alignment along a common coordinate system (Rohlf and Slice 1990) resulting in the removal of the extraneous information of landmark´s size and orientation (Savriama 2018). GM are powerful analytic tools in constant development that offer a new way of studying species evolution (Savriama 2018), systematics (Liu et al. 2018; Menini Neto et al. 2019), and even phylogeography (Terral et al. 2004, 2012) or ecology (García-Jain et al. 2022) and archaeophenomics (Evin et al. 2022) by collecting and comparing the morphology of organisms. GM studies in plants have been implemented with ancient plant organs (Terral et al. 2004, 2012), functional traits (Van der Niet et al. 2010; Neustupa and Nemcova 2022), and floral symmetry (Chen et al. 2018; Savriama 2018).

*Carex* L. (Cyperaceae) with more than 2000 species, is one of the five largest genera among angiosperms and one of the two largest within monocots (Govaerts et al. 2022). The genus is largely adapted to temperate-cold climates and has its origin in the Late Eocene (c. 37 mya), probably in southeast Asia from where it spread, reaching currently a nearly cosmopolitan distribution (Martín-Bravo et al. 2019). Traditional taxonomic treatments of *Carex*, as in most plant groups, have been primarily based on morphological data. These classifications’ frameworks are known to be affected by morphological homoplasy, which has blurred the systematic relationships among species groups (Jiménez-Mejías et al. 2016a). Among the organs used in the taxonomic delimitation of *Carex*, the utricle is by far the most relevant. It is a bract-derived organ that is modified into a false fruit enclosing the nutlet (see Jiménez-Mejías et al. 2016b). Its morphology is usually conserved among the species of the different natural groups within the genus (see Roalson et al. 2021). All taxonomic treatments of *Carex* use the utricle to circumscribe and identify groups (e.g. Chater 1980; Egorova 1999; Ball and Reznicek 2002; Luceño 2008; Dai et al. 2010).

**Figure 1.**
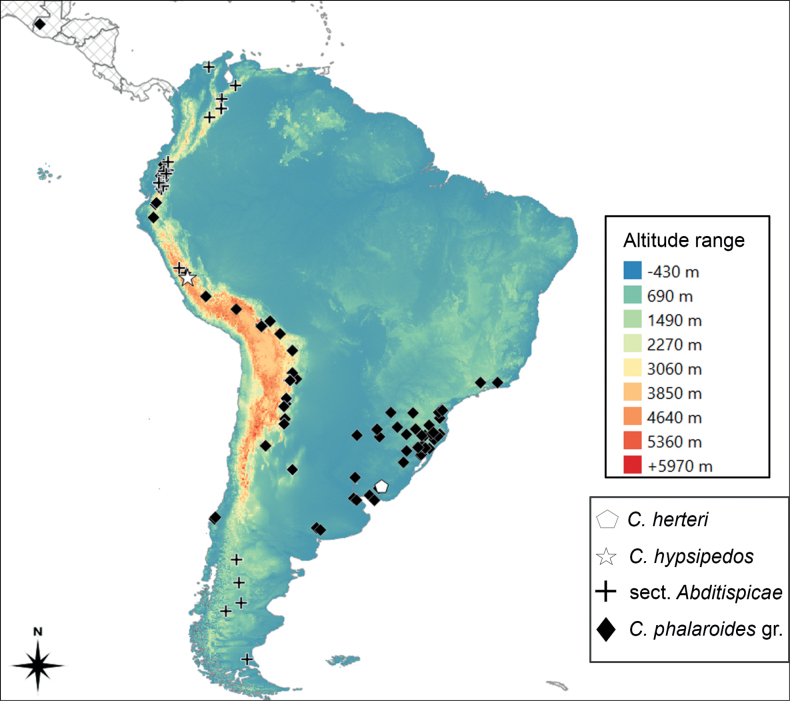
South America elevation map showing known distribution of the taxa considered in this study.

**Figure 2.**
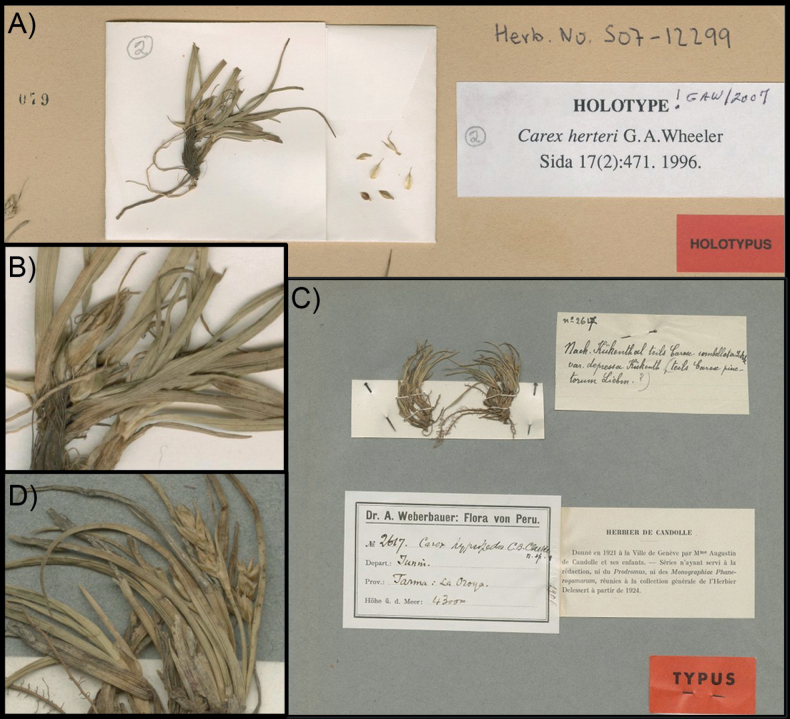
Images of the problematic species type collections **A** holotype of *C.herteri* (Herter 19091, S) **B** zoom in of holotype of *C.herteri* (Herter 19091, S) **C** holotype of *C.hypsipedos* (Weberbauer 2617, G) **D** zoom in of holotype of *C.hypsipedos* (Weberbauer 2617, G).

According to Jiménez-Mejías (2017), about 200 species of *Carex* are native to South America, most of them endemic to the continent where they are mostly restricted to temperate-cold environments such as steppes, Patagonian forests and pampas and, in tropical latitudes, mountainous areas. An abnormally high number of *Carex* species at these areas exhibit dwarfism, with strong character reduction and acaulescency (Jiménez-Mejías et al. 2021). Such modifications result in diminutive plants with similar homoplasic morphological appearances. This is the case of two South American species, *C.herteri* G.A.Wheeler, an endemic to the pampas of Uruguay, and *C.hypsipedos* C.B.Clarke from the dry Andes of central Peru (Wheeler 1996; Poindexter et al. 2017; Fig. 1). Both species are only known from their type collections, from 1920 and 1906 respectively (Fig. 2).

To date, *Carexherteri* and *C.hypsipedos* have been included in the group of *C.phalaroides* Kunth (hereafter *C.phalaroides* gr.; subg. Psyllophorae, sect. Junciformes) due to superficial morphological similarities (Wheeler 1996; Poindexter et al. 2017). The *C.phalaroides* gr. is a taxonomic complex of four to six species depending on the treatment, morphologically characterized by stems usually well-developed, sometimes acaulescent, pedunculate bisexual (androgynous) spikes, utricles with a short beak and an indumentum of hairs or papillae, and three stigmas (Hoff-Silveira and Longhi-Wagner 2012; A.M.A. and P.J.-M pers. obs). It is a Neotropical group (Fig. 1) which mainly inhabits temperate and subtropical latitudes of South America, although it reaches tropical areas northwards along the Andes, displaying isolated occurrences in the Central American Cordillera. The *C.phalaroides* gr. species are ecologically atypical among Neotropical sedges as they primarily occur in temperate and subtropical habitats, such as the Atlantic forest and pampas (Benítez-Benítez et al. 2021). Despite their initial attribution to the *C.phalaroides* gr., both *C.herteri* and *C.hypsipedos* display characters that would be deviant within it. On the one hand, *C.herteri* differs from *C.phalaroides* gr. species in its utricle size, presence of a conspicuous beak, and lack of indumentum (Wheeler 1996). On the other hand, *C.hypsipedos* diverges from *C.phalaroides* gr. taxa in the number of stigmas, as it has only two instead of three, and also the utricle with a conspicuous beak (Poindexter et al. 2017). Therefore, its consideration as part of the *C.phalaroides* gr. is still tentative and pending confirmation.

Among all the remaining South American *Carex* groups, the only other alternative match for these two species would be Carexsect.Abditispicae G.A.Wheeler (subg. Carex). Section Abditispicae comprises a group of eight species endemic to South America (Roalson et al. 2021). Its taxa are characterized by acaulescent habit, with lateral female spikes borne at or near the plant base, often hidden among leaves but sometimes with well-developed peduncles, terminal male spike or androgynous, utricles with an indumentum more or less papillose, and with a truncate beak, and two stigmas (Wheeler 1987). This group primarily inhabits Patagonia and Tierra del Fuego, although a few species reach the Tropic of Capricorn and further north through the Andes (Wheeler 2002). The section typically inhabits areas such as moist or wet grasslands, bofedales (Andean bogs), swamps, lake shores, and wet sands and gravels by the sea (Wheeler 1987). The frequent dwarf size of sect. Abditispicae species, which makes its collection difficult by non-specialists, in addition to the remote areas they inhabit, might be the cause of the poor representation of species of this group in herbaria (Jiménez-Mejías et al 2023), with some species known only from a handful of collections or only from their type ones (Wheeler 1987, 1996, 2002).

The taxonomic placement of *C.herteri* and *C.hypsipedos* as part of the *C.phalaroides* gr. should be considered tentative, due to the manifest character reduction of the two taxa, the frequent morphological homoplasy in *Carex*, their reported differences with the ascribed group, and the lack of molecular data. Alternatively, sect. Abditispicae seems to be a reasonable competitive group for the systematic adscription of the two species. In this study we aim to re-evaluate the attribution of *C.herteri* and *C.hypsipedos* to either *C.phalaroides* gr. or sect. Abditispicae in absence of available molecular data by analyzing the utricle, an organ of paramount taxonomic relevance in *Carex* together with other morphological characteristics using GM, a tool barely used for taxonomic delimitation in Cyperaceae, and traditional morphometrics, respectively.

## ﻿Materials and methods

### ﻿Geometric morphometric sampling

**Figure 3.**
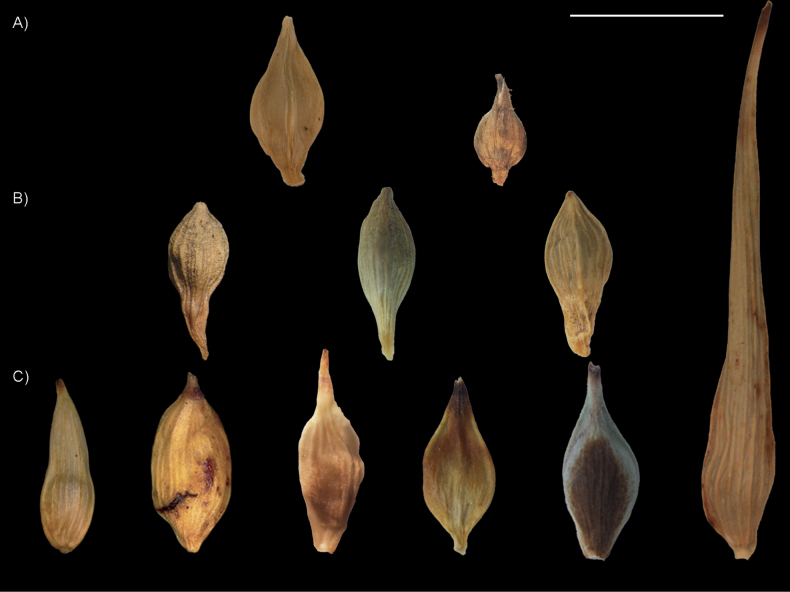
Representative utricles images used for GM analyses. From the top to the bottom, left to right **A** problematic species: *Carexherteri* (Herter, W.G.F., 19091, S), *C.hypsipedos* (Weberbauer 2617, G) **B***C.phalaroides* gr.: *C.gibertii* (Arechavaleta *s.n.*, US), *C.paraguayensis* (L. Pereira-Silva 350, FLOR) and *C.phalaroides* (G. Rodríguez-Palacios 23GERP15, UPOS) **C**C.sect.Abditispicae: *C.acaulis* (DM Moore 1240, K), *C.collumanthus* (PJ Grubb 339, K), *C.humahuacaensis* (S. Martín-Bravo et al., 178SMB21, UPOS), *C.ruthsatzae* (G. Rodríguez-Palacios 46GERP15, UPOS), *C.subantarctica* (Marcia Waterway, MW2015.020, UPOS), and *C.macrosolen* (S. Martín-Bravo et al., 11SMB10, UPOS). Scale bar: 4 mm.

We selected utricles from 11 species (Fig. 3; Suppl. material 1): all four of *C.phalaroides* gr. (representatively covering its entire known morphological and geographical variation), and six from sect. Abditispicae (80% of the known species; Roalson et al. 2021). Due to the scarcity of sect. Abditispicae herbarium collections (and in particular of specimens bearing ripe utricles), we had to limit our sampling to the few mature specimens we located. After studying *in situ* the full collections of K, QCA, QCNE, and UPOS herbaria, we only managed to obtain 15 utricles: two from *C.acaulis*; three from *C.collumanthus*; one of *C.humahuacaensis*; two of *C.macrosolen*, four from *C.ruthsatzae*, and three of *C.subantarctica*. For *C.phalaroides* gr. we managed to obtain 32 utricle images: 14 of *C.gibertii*, three of *C.moesta*, three of *C.paraguayensis*, and 12 of *C.phalaroides* (Suppl. material 1). Detailed pictures of the utricles were taken with a Nikon stereoscopic microscope (Nikon SMZ745) and an Olympus stereoscopic microscope (Olympus SZX16). We compiled a set of 49 2D-scaled images.

### ﻿Geometric morphometric analyses

Nine fixed landmarks were placed on homologous points within utricles following Jiménez-Mejías and Martinetto (2013) approach to the carpological features of *Carex*. All these fixed landmarks were limited to the beak and utricle base, as homologous structures between species (Suppl. material 2: fig. S1). In addition, to collect as much shape information as possible from the utricle body, we placed eight semi-landmarks equidistantly, on non-homologous points of the utricle body margins. These defined two curves on the right and left side of the utricle, from the base of the beak to the beginning of the substipitate base. Landmarks were digitalized, on images using TPSDig2 (Rohlf 2015) and datasets were created in a .tps format. All fix and sliding landmarks were positioned in the same order in all images.

Main GM analysis was conducted with a complete dataset including all 49 utricle images (from hereinafter referred as “complete GM dataset”). We split this dataset into sect. Abditispicae species (15 utricle images) and *C.phalaroides* gr. species (32 utricle images), creating *Abditispicae*GM dataset and *C.phalaroides*GM dataset two and three, respectively. Datasets two and three excluded problematic species thus we obtained the consensus utricle configuration for each group to allow visual shape comparison with the problematic species. GM analysis was performed four times, first for identifying potential outliers through a PCA scatter plot, second for the complete GM dataset, and third and fourth for shape exploration of sect. Abditispicae and *C.phalaroides* gr. datasets, respectively. The GM analysis of the complete dataset revealed that the highly deviant utricle of *C.macrosolen* induced a strong bias to the analysis generating a substantial deviation to the PCA, due to its large peak size (Suppl. material 2: fig. S2), thus, this was removed from all subsequent GM analyses, leaving the complete dataset with only 47 utricle images.

We proceeded to landmark analysis with geomorph, R package v.4.0.2 (Adams et al. 2021). Semilandmarks were set as sliding points with the *geomorph*::*define.sliders* function. We subsequently performed generalized Procrustes analysis (GPA) using the minimized squared distances method. GPA calculates the consensus configuration of the dataset, along with its shape variation, and makes a separation within size and shape components of the datasets (Viscosi and Cardini 2012). It is here used as a superimposition method because it has been shown to be accurate in sample means estimation (Rohlf 2000a, b, 2003). GPA was performed with the *geomorph*::*gpagen* function and a maximum of 1000 iterations. The studied utricle morphospace was visualized by performing a principal component analysis (PCA) only for the complete dataset, and the shape variation within the morphospace was depicted with *geomorph*::*picknplot.shape* function. Subsequently, a Procrustes multivariate analysis of variances MANOVA was carried out with the *geomorph*::*procD.lm* function to assess statistical patterns of shape variation for a set of Procrustes aligned coordinates between the considered groups (Adams et al. 2021). Accounting for the small size of our dataset, the significance of shape variation between the two major groups was performed against a null model generated by permuted resampling, which uses a residual randomization permutation of 999 replicates (Collyer et al 2015; Renner et al 2018). The mean shape of all sampled utricles for every dataset was visualized with *geomorph*::*mshape* and *geomorph*::*plotRefToTarget* functions. To achieve an objective attribution of the problematic species to either sect. Abditispicae or *C.phalaroides* gr. we performed a Discriminant Function Analysis (DFA), more precisely the Linear Discriminant Analysis (LDA) with MASS R package (Venables and Ripley 2002). For this analysis we set a train dataset only with sect. Abditispicae and *C.phalaroides* gr. species and prepared two tests, one for *C.herteri* and another for *C.hypsipedos*. These datasets were created from coordinates calculated in the GPA. We checked the coordinates of *C.herteri* and another for *C.hypsipedos* correctly and incorrectly assigned to each of the groups of train dataset through confusion matrices.

### ﻿Traditional morphometric analyses sampling

Characters to be measured for *C.phalaroides* gr. initially followed the review of *Carex* in Rio Grande do Sul (Hoff-Silveira and Longhi-Wagner, 2012) with slight modifications according to our own observations. The different structures to be measured were selected from the middle zone of each organ, aiming for homogeneity within the data and to facilitate the comparison between individuals, with the following exceptions (1) the bracts, in which we selected the upper spike bract and the lower spike bract and (2) the length and width of the leaves, for which the longest and the widest leaves of each individual were selected, respectively. A list of 38 potentially diagnostic characters was established, although exploratory PCA analyses retrieved that only 24 characters were diagnostic. Our final dataset was constituted by 24 morphological characters measured on 56 individuals (Suppl. material 3).

Sampling of sect. Abditispicae relied on literature data in order to take into account the entire variation span known for each taxon (Suppl. material 3) after contrasting that our own measurements felt within the reported variation intervals.

### ﻿Principal component analysis

Principal component analysis was carried out on Rstudio v. 1.4.1717 (R Core Team 2022) using 11 morphological variables: two vegetative characters and the rest reproductive-related characters (Table 1) as these were the only characters available for every taxa. For every character in all the considered taxa of sect. Abditispicae we included three independent data as detailed on the corresponding taxonomic description (Suppl. material 3): maximum, minimum and the mean. This way we ensured the consideration of the maximum possible span of each species within the morphospace.

**Table 1.** Summary of the morphological traits analyzed in the two different PCA carried out in this study.

Morphological traits for *Carex* sect. *Abditispicae – Carexphalaroides* gr. PCA	
**Organ**	**Character**
Leaf	Length (mm)
Leaf	Width (mm)
Lower spike bract	Width (mm)
Inflorescence	Length (mm)
Spike	Length (mm)
Spike	Width (mm)
Scale	Length (mm)
Utricle	Length (mm)
Utricle	Width (mm)
Achene	Length (mm)
Achene	Width (mm)

### ﻿Mean comparatives and non-parametric tests

The significance of those characters that allowed the best separation of each of the two morphogroups (*C.phalaroides* gr. and sect. Abditispicae; see results) was evaluated by non-parametric Kruskal–Wallis test with Rstudio v. 1.4.1717 (R Core Team 2022). After discarding that the dataset had a normal distribution, we employed Kruskal-Wallis test as a non-parametric alternative to ANOVA test. As visual support for the test, we performed violin graphic plots to present the comparison between the problem species and the morphogroups we tested them against.

## ﻿Results

### ﻿Geometric morphometrics analyses

**Figure 4.**
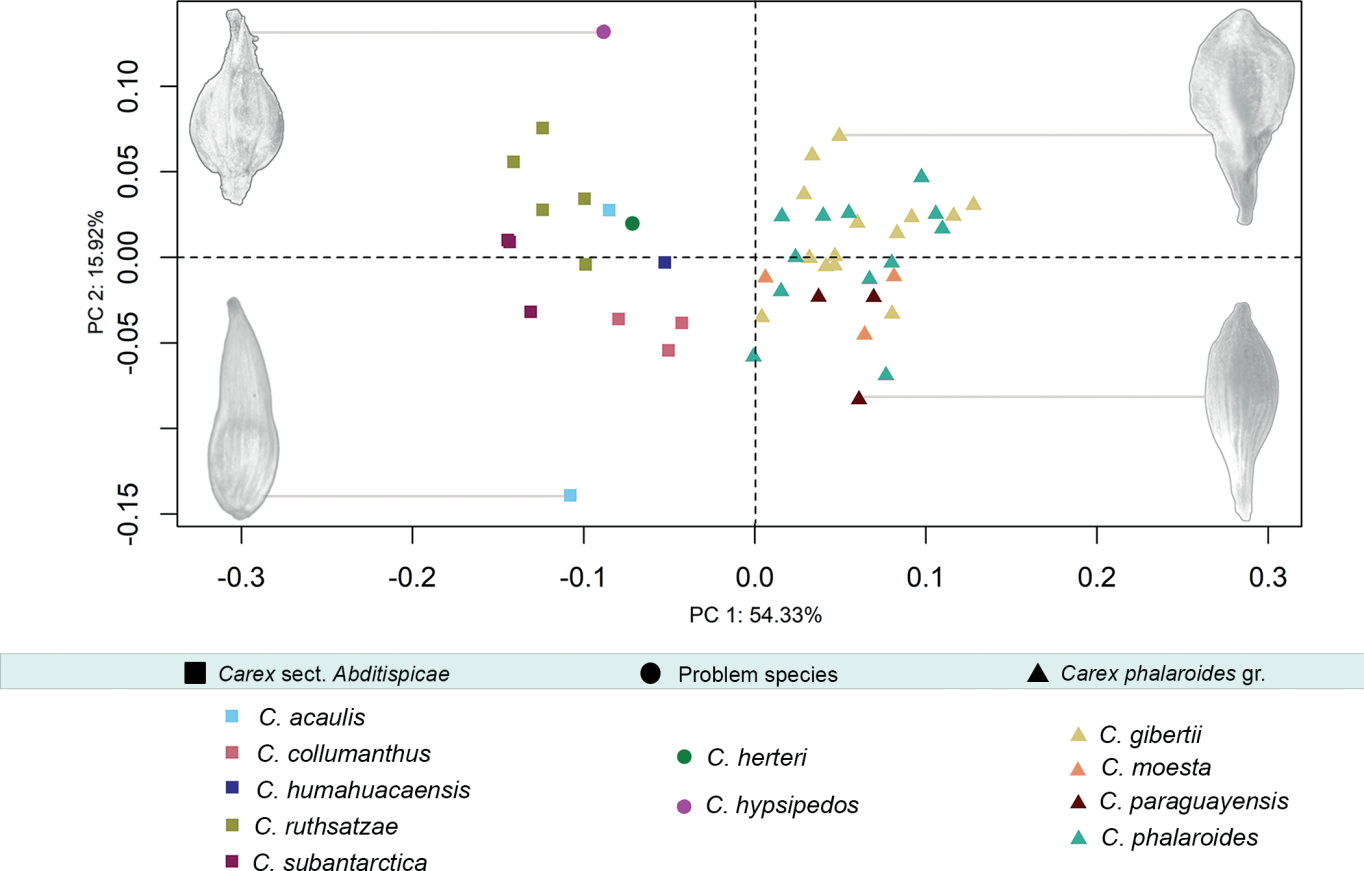
PCA scatter-plot of the geometric morphometric analysis excluding *Carexmacrosolen*. Squares represent sect. Abditispicae taxa, triangles represent *C.phalaroides* gr. taxa, and circles represent *C.herteri* and *C.hypsipedos* taxa. Utricles shapes at the margins of the graph display the extreme shapes of the morphospace for a better visualization of the utricle morphological features with greater weight within the principal components.

**Figure 5.**
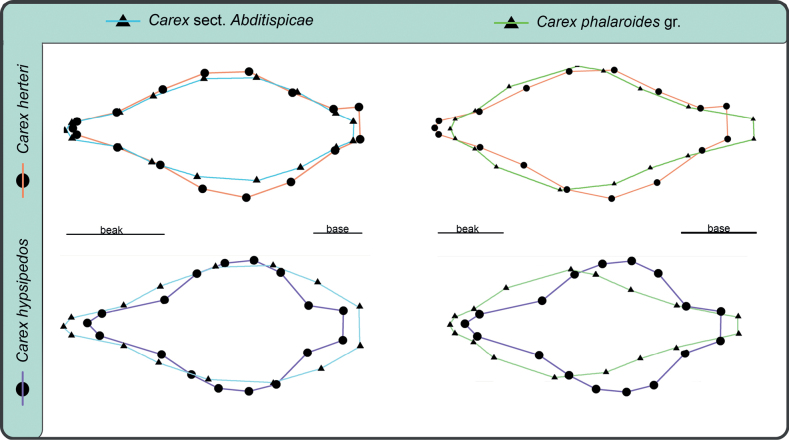
Comparative figures of consensus utricle shapes of both Carexsect.Abditispicae (triangles and blue line) and *C.phalaroides* gr. (triangles and green line) with *C.herteri* (circles and orange line) and *C.hypsipedos* (circles and purple line).

Procrustes analyses performed for the different datasets recovered the consensus utricle configurations and deviations for every landmark and semilandmark coordinate (Suppl. material 2: fig. S3). The low utricle shape affinities of the two tested groups were compared and shown at Suppl. material 2: fig. S4. Subsequently, MANOVA results show shape variation is significantly different between the two considered groups (Table 2A) denoting that these are well delimited and can be successfully differentiated using the proposed configuration of landmarks and semilandmarks. For the PCA of the complete dataset, the first three principal components accumulated up to 81.2% of the variance (54.3%, 17%, 10%). PCA scatter-plot for the complete dataset displays the position of the problematic species, *C.herteri* and *C.hypsipedos*, within the morphospace (Fig. 4), illustrating a higher proximity to the sect. Abditispicae cluster than to *C.phalaroides* gr one. In the case of *C.herteri*, its affinity was much clearer than for *C.hypsipedos*. LDA model only obtained one linear discriminant and prior probabilities for the main groups were 0.347 for sect. Abditispicae and 0.653 for *C.phalaroides* gr. The model obtained a 0.959 of accuracy meaning 95.9% of samples were correctly classified (Table 2 B), only four coordinate samples were misclassified. When testing *C.herteri* dataset in the trained model, both of its coordinates were placed within sect. Abditispicae with an accuracy = 1, while *C.hypsipedos* shape information was not clearly positioned in either of the two groups, so the accuracy of the model in this case was only 0.5. Visually, the four graphs comparing the consensus configurations of the sect. AbditispicaeGM and *C.phalaroides* gr. GM datasets with the problematic species shapes (Fig. 5) assist the results revealed by PCA and DFA analyses of higher shape affinity of *C.herteri* with sect. Abditispicae than with *C.phalaroides* gr., while shape resemblance of *C.hypsipedos* remained uncertain.

**Table 2.** Results of GM statistical analyses.

A) MANOVA test summary table							
	d.f.	Sum Sq	Mean Sq	R Sq	F value	Z	Pr(>F)
Groups	1	0.26965	0.269650	0.46926	41.555	4.8696	9.999e-05
Residuals	47	0.30498	0.006489	0.53074	–	–	–
Total	48	0.57463	–	–	–	–	–
**B) Summary of linear discriminant analyses results showing confusion matrices of predicted classes of test dataset within train dataset and its accuracy**							
Confusion matrix train dataset		Sect. Abditispicae	*C.phalaroides* gr	Confusion matrix test dataset		New data	
				Predicted		*C.herteri*	*C.hypsipedos*
Sect. Abditispicae		31	1	Sect. Abditispicae		2	1
*C.phalaroides* gr		3	63	*C.phalaroides* gr		0	1
Accuracy		0.959		Accuracy		1	0.5

### ﻿Traditional morphometric analyses

**Figure 6.**
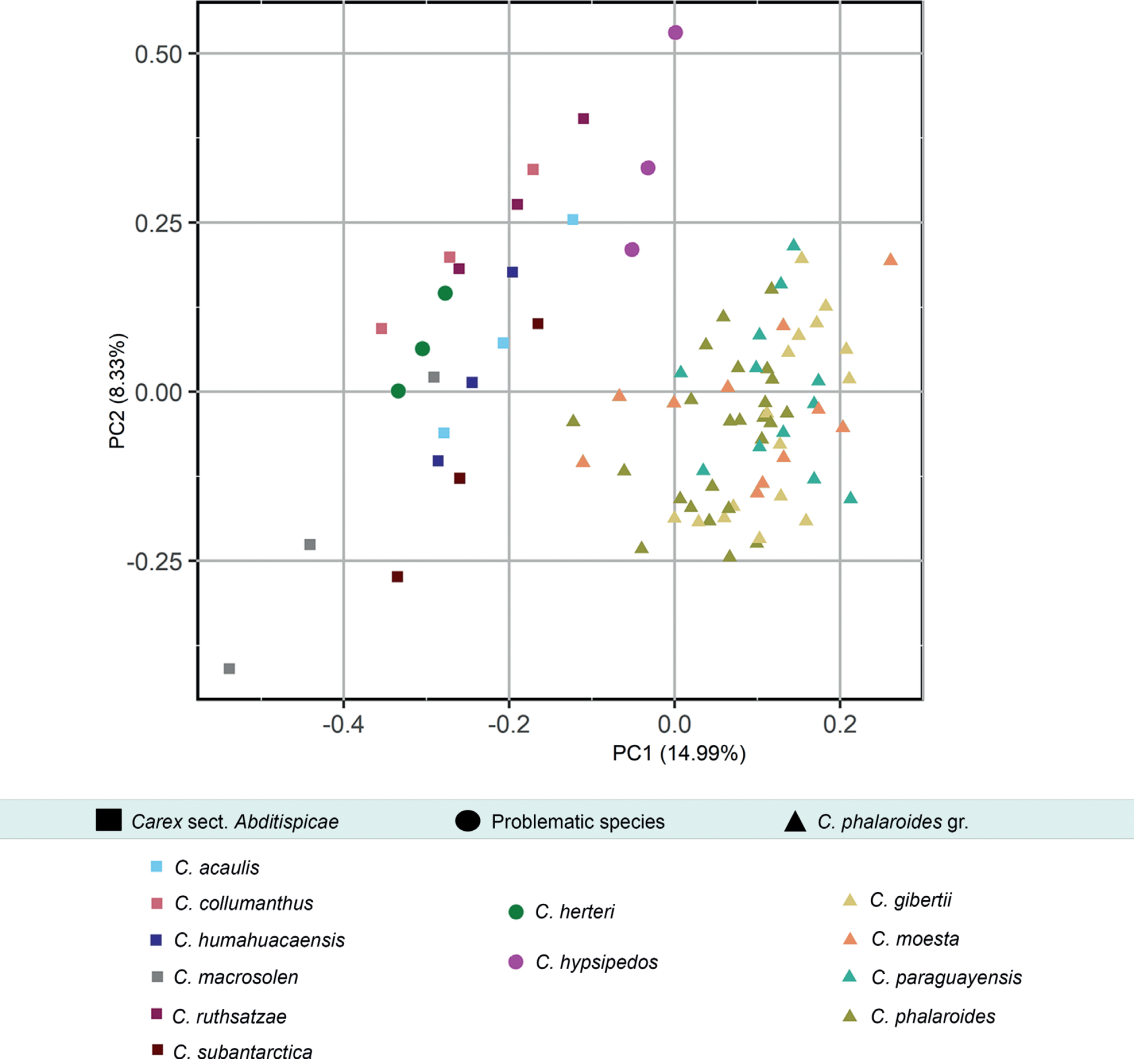
PCA scatter-plot of the traditional morphometric analysis. Squares represents sect. Abditispicae taxa, circles represent *C.herteri* and *C.hypsipedos*, and triangles represents *C.phalaroides* gr. taxa.

**Figure 7.**
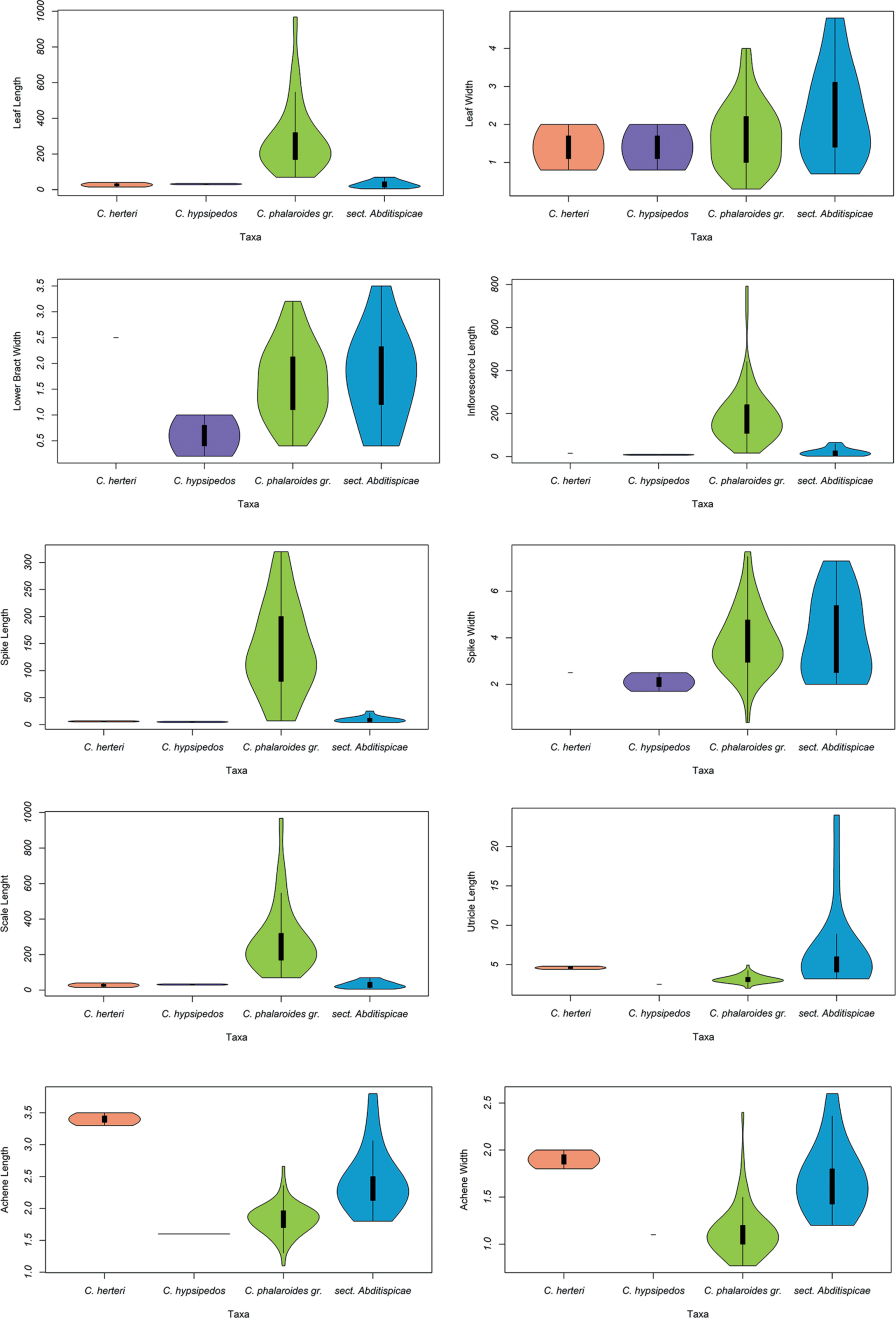
Violin plots; Violin plots illustrating distribution and mean differences for the analyzed characters with traditional morphometrics for the problematic species, *C.phalaroides* gr. and sect. Abditispicae.

PCA performed to assess *C.herteri* and *C.hypsipedos* proximity to major groups included all the available morphological characters (Table 1), since these already allowed the best separations of morphogroups. We only retained principal components with eigenvalues>1. The first two principal components accumulated the 65.9% of the variance and the 75.5% on the first three: PC1 43.1%; PC2 22.8%; PC3 9.7%. PCA values for every character are shown at Table 3. PCA scatter-plot PC1–PC2 (Fig. 6) revealed the existence of two separated clusters, one for sect. Abditispicae and another for *C.phalaroides* gr. The problematic species were placed close to the first one, indicating morphological similarities for the analyzed characters. The morphological characters which contributed the most to the first principal components were leaf length, utricle length and width and achene length and width, while to the second component important characters were the lower spike bract width, leaf width, spike width and inflorescence length (Table 3). For the Kruskal–Wallis test, eight of the eleven analyzed characters obtained a significant *p*–value < 0.05 (Table 3) . Only two of them were vegetative characters (leaf and lower spike bract lengths) while the remaining were reproductive-related characters. Most significant p-values were scored by leaf length (3.513e-11), utricle width (4.062e-09), utricle length (1.987e-09), achene length (2.935e-08) and achene width (1.218e-07). Violin plots at Fig. 7 illustrate the distribution differences of the measurement dataset supporting the use of non–parametric tests to assess mean differences between groups.

**Table 3.** PCA and non-parametric tests results. First two principal component values resulting from the PCA performed for the traditional morphometric study. The *p*–values from the Kruskal-Wallis test are also indicated (* indicate significant results).

Morphological traits	PC1	PC2	Kruskal-Walis test
Leaf length (cm)	0.370981181	-0.30144017	3.513e-11***
Leaf width (mm)	0.128838175	-0.44318792	0.3196
Lower spike bract width (mm)	0.081672924	-0.46994517	0.01402*
Inflorescence length (cm)	0.29404183	-0.36809907	6.513e-11***
Spike length (cm)	0.338174775	-0.33235128	7.273e-10***
Spike width (mm)	0.006355108	-0.3702008	0.0221*
Scale length (mm)	-0.22673691	-0.18833859	0.4813
Utricle length (mm)	-0.37000759	-0.18041262	1.987e-09***
Utricle width (mm)	-0.3915743	-0.04835903	4.062e-09***
Achene length (mm)	-0.38158338	-0.12137059	2.935e-08***
Achene width (mm)	-0.38857237	-0.15419903	1.218e-07***

## ﻿Discussion

### ﻿Novel data shed light on the systematic affinities of the two problematic species

Dwarf species *Carexherteri* and *C.hypsipedos* were assigned to *C.phalaroides* gr. by Wheeler (1996) and Poindexter et al. (2017) respectively. This designation was based on morphological affinities not tested under statistical approaches. Given the morphological complexity of the group, its adscription was in need of a revision. Carexsect.Abditispicae was a major candidate to incorporate these species as these concur on distribution and morphological characteristics (Wheeler 1987, 1989, 2002).

Our GM, DFA and traditional morphometrics results reveal a high statistical support and a close utricle shape resemblance among sect. Abditispicae and *C.herteri* for all the analyses performed (Figs 2–4), therefore this species may be better considered as part of this section based on its morphological features. Contrastingly, *C.hypsipedos* does not display evident statistical affinities, or shape resemblance with either sect. Abditispicae or *C.phalaroides* gr. so its affiliation persists unsolved, though it can be excluded as a member of the *C.phalaroides* gr.

Nevertheless, it would be desirable confirmation from procedures such as DNA barcode for two main reasons: (1) The frequent morphological homoplasy that affects the delimitation of infrageneric units within the genus (Jiménez-Mejías et al. 2016a), exacerbated in this particular case by the extreme reduction of such plants (dwarfism, see Jiménez-Mejías et al. 2021), that might further confound their morphological affinities; and (2) The extremely low sampling size of the problematic species (known only from their type collections), thus perhaps no representative of the entire species variation. Ripe utricle morphology is usually fairly constant, as supported by its recurrent use in identification keys (e.g. see keys in Egorova 1999 or Ball and Reznicek 2002). However, variation in utricle size, and also moderately in shape, is also known in *Carex* (Jiménez-Mejías et al. 2017, 2018). Accordingly, we cannot entirely rule out that the included problematic taxa samples were outliers and so could be somehow biasing the inferred affinities of the two problematic taxa, although it would be certainly unexpected because of the consistency in shape variation within each of the detected groups. Dwarfism, acaulescency, and character reduction should be considered the principal cause of the deficiency of herbarium collections and the absence of field citations of these two problematic species, due to their inconspicuousness. Due to the impossibility of performing a destructive sampling on the already poor type collections from which *C.herteri* and *C.hypsipedos* were described, the knowledge of these two taxa would benefit from a focused fieldwork sampling.

The adscription of *C.herteri* to sect. Abditispicae would imply an area extension of a thousand km from the Patagonian steppes and high mountainous Andean habitats of the section to the Uruguayan Pampa. This, in turn, implies a much wider ecology for the group, from the cold-dry steppes and high-altitude habitats of the known species to the warm dryness of the pampa. As a common factor, Carexsect.Abditispicae ecology seems to be linked to stressful environments and may behave as pioneers in colonization processes.

### ﻿Utility of geometric morphometrics in testing systematic affinities in graminoids

Our approach using GM has assessed fruit shape variation in a non–qualitative way, as it is commonly studied on traditional morphometrics (Chen et al. 2018). Some examples of systematic and taxonomic implications derived from GM have been previously done in Liu et al. (2018) with Chinese oaks leaves, Terral et al. (2012) with the seeds of *Phoenix* genera species, or Van der Niet et al. (2010) assessing flower shape variation. These studies agreed in the application of GM as a useful approach for providing detailed information on the morphological variation of the plant structures with taxonomic value. In addition, research on plant organ shapes and its relationship with other organisms or environmental factors might shed additional light on other fields such biogeography, ecology, and genetics, as we also do when we assign *C.herteri* to sect. Abditispicae.

Our study supports the utility of GM on testing systematic affinities in species with graminoid morphology, particularly for Cyperaceae. To this end we used carpological characters, which have been often ignored in sedges, despite the useful characters for group delimitation residing in such organs (Jiménez-Mejías and Martinetto 2013). Successful differentiation between and within complicated groups such as sect. Abditispicae and *C.phalaroides* employing utricle shape sets a landmark for future taxonomic studies in a genus where its general morphology is typically affected by homoplastic processes.

## ﻿Conclusions

Utricle shape variation along with other morphological features analyzed with GM and traditional morphometric approaches, respectively, support the exclusion of these two species from their traditionally affiliated group (*C.phalaroides* gr.). Moreover, *C.herteri* show clear affinities to sect. Abditispicae for both approaches. Besides, *C.hypsipedos* remains an *incertae sedis* species as it did not show affinities with any of these groups, thus further studies are needed for these taxa. Additionally, we employ for the first time geometric morphometrics tools and show its potential utility to approach the systematic affinities of taxo­nomically problematic sedge species.

## ﻿Additional information

### Author ORCIDs

Ana Morales-Alonso https://orcid.org/0000-0003-1497-1070

Tamara Villaverde https://orcid.org/0000-0002-9236-8616

Pedro Jiménez-Mejías https://orcid.org/0000-0003-2815-4477

### Data availability

All of the data that support the findings of this study are available in the main text or Supplementary Information.

## Supplementary materials

Supplementary material 1 GM material Data type Excel spreadsheet Explanation note Material used in this work for the utricle images. Taxon, botanical country (Brummitt 2001), locality, collection information (herbarium or collector code) and number of utricles is provided for each sample. https://binary.pensoft.net/file/910117This dataset is made available under the Open Database License (http://opendatacommons.org/licenses/odbl/1.0/). The Open Database License (ODbL) is a license agreement intended to allow users to freely share, modify, and use this Dataset while maintaining this same freedom for others, provided that the original source and author(s) are credited.Ana Morales-Alonso, Tamara Villaverde, Pedro Jiménez-Mejías

Supplementary material 2 Utricle slide with landmarks; PCA plot with *C.macrosolen*; Consensus configurations; Group comparison Data type PDF file; fig. S1–S4 Explanation note Representation of landmark placement within the utricle. Green dots represent landmarks and blue dots semi-landmarks. From the top to the bottom, left to right. A) Problematic species: *Carexherteri* (Herter, W.G.F., 19091, S), *C.hypsipedos* (Weberbauer 2617, G). B) *C.phalaroides* gr.: *C.gibertii* Arechavaleta s.n.US), *C.paraguayensis* (L. Pereira-Silva 350, FLOR) and *C.phalaroides* (G. Rodríguez-Palacios 23GERP15, UPOS). C) C.sect.Abditispicae: *C.acaulis* (DM Moore 1240, K), *C.collumanthus* (PJ Grubb 339, K), *C.humahuacaensis* (S. Martín-Bravo et al. 178SMB21, UPOS), *C.ruthsatzae* (G. Rodríguez-Palacios 46GERP15, UPOS), *C.subantarctica* (Marcia Waterway MW2015.020, UPOS) and *Carexmacrosolen* (S. Martín-Bravo et al., 11SMB10, UPOS) utricle was finally excluded from the analysis as it induced a strong bias. Scale bar of 4mm. PCA scatter-plot of the geometric morphometric analysis including *C.macrosolen*. Squares represents sect. Abditispicae taxa, triangles represent *C.phalaroides* gr. taxa, and circles represent *C.herteri* and *C.hypsipedos* according to the figures legend. Utricles shapes at the margins of the graph display the extreme shapes of the morphospace for a better visualization of the utricle morphological features with greater weight within the principal components. Comparative figure of consensus configurations between datasets: A) representing the utricle consensus configuration (black dots and grey line) and deviations from it (orange dots) for the complete dataset. B) representing the utricle consensus configuration (black dots and blue line) and deviations from it (blue dots) for the sect. Abditispicae dataset. C) representing the utricle consensus configuration (black dots and green line) and deviations from it (green dots) for the *C.phalaroides* gr. dataset. Comparative figure for the consensus utricle configuration of *C.phalaroides* gr. (green triangles) with sect. Abditispicae (blue dots). https://binary.pensoft.net/file/910118This dataset is made available under the Open Database License (http://opendatacommons.org/licenses/odbl/1.0/). The Open Database License (ODbL) is a license agreement intended to allow users to freely share, modify, and use this Dataset while maintaining this same freedom for others, provided that the original source and author(s) are credited.Ana Morales-Alonso, Tamara Villaverde, Pedro Jiménez-Mejías

Supplementary material 3 Traditional morphometrics material Data type Excel spreadsheet Explanation note Material used in this work for traditional morphometric analyses. A: measured specimens of *C.phalaroides* gr. Taxa are indicated, the botanical country (Brummitt 2001), the locality and an identification code (herbarium or collector code); B: list the taxonomic treatments from where the representative measurements of sect. Abditispicae species were taken. https://binary.pensoft.net/file/910119This dataset is made available under the Open Database License (http://opendatacommons.org/licenses/odbl/1.0/). The Open Database License (ODbL) is a license agreement intended to allow users to freely share, modify, and use this Dataset while maintaining this same freedom for others, provided that the original source and author(s) are credited.Ana Morales-Alonso, Tamara Villaverde, Pedro Jiménez-Mejías
