# Supplementary material for: ﻿Geometric Morphometrics sheds light on the systematics affinities of two enigmatic dwarf Neotropical sedges (Carex, Cyperaceae)
Source: PhytoKeys. 2023 Sep 21;232:167–87. doi: 10.3897/phytokeys.232.100410 (PMC10540071; doi:10.3897/phytokeys.232.100410)

Figure S1. Representation of landmark placement within the utricle. Green dots represent landmarks and blue dots semilandmarks. From the top to the bottom, left to right. A) Problematic species: *Carex herteri* (Herter, W.G.F., 19091, S), *C. hypsipedos* (Weberbauer 2617, G). B) *C. phalaroides* gr.: *C. gibertii* Arechavaleta *s.n.*US), *C. paraguayensis* (L. Pereira-Silva 350, FLOR) and *C. phalaroides* (G. Rodríguez-Palacios 23GERP15, UPOS). C) *C.* sect. *Abditispicae*: *C. acaulis* (DM Moore 1240, K), *C. collumanthus* (PJ Grubb 339, K), *C. humahuacaensis* (S. Martín-Bravo et al. 178SMB21, UPOS), *C. ruthsatzae* (G. Rodríguez-Palacios 46GERP15, UPOS), *C. subantarctica* (Marcia Waterway MW2015.020, UPOS) and *Carex macrosolen* (S. Martín-Bravo et al., 11SMB10, UPOS) utricle was finally excluded from the analysis as it induced a strong bias. Scale bar of 4mm.

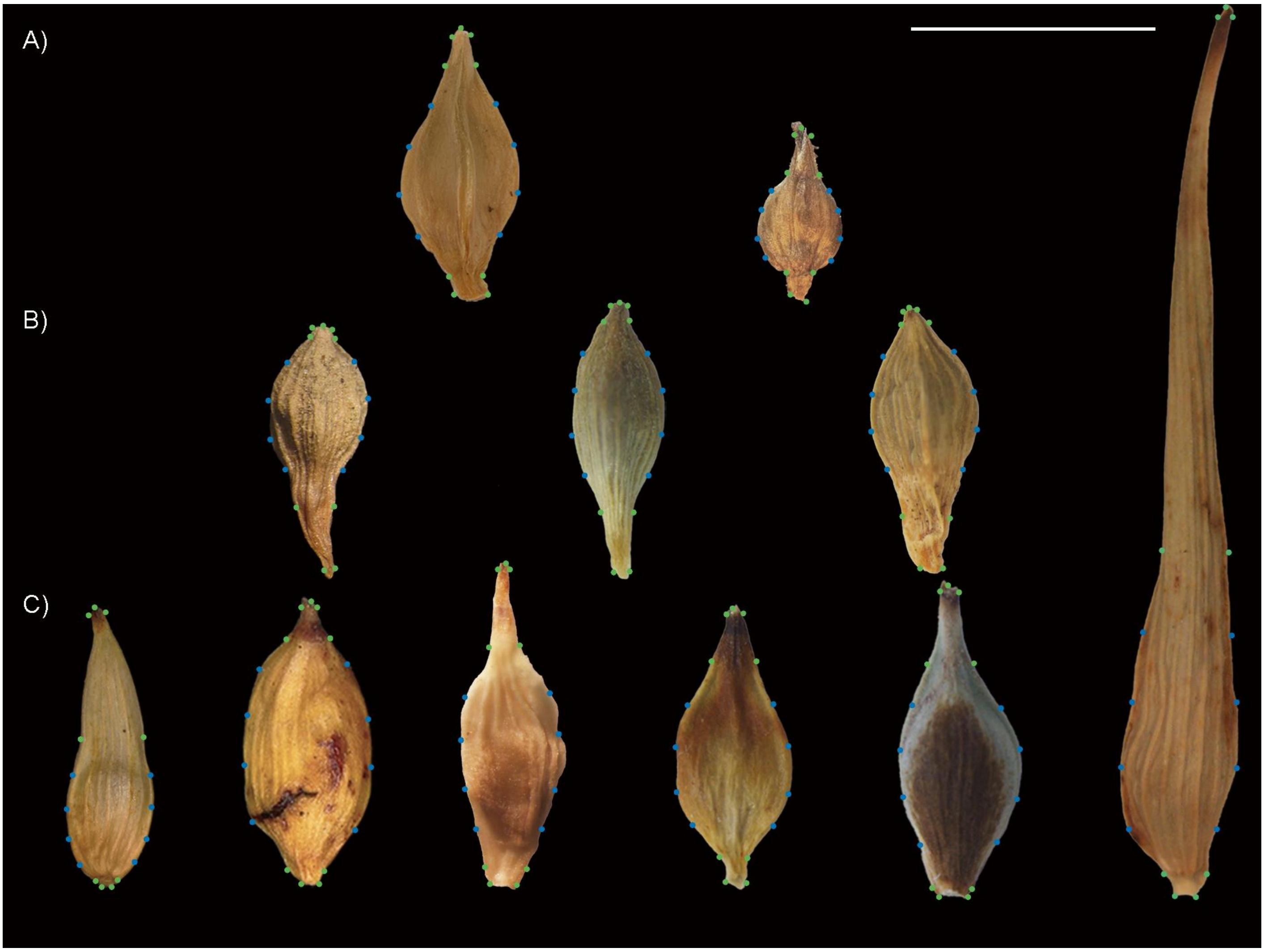

Figure S2. PCA scatter-plot of the geometric morphometric analysis including *C. macrosolen*. Squares represents sect. *Abditispicae* taxa, triangles represent *C. phalaroides* gr. taxa, and circles represent *C. herteri* and *C. hypsipedos* according to the figures legend. Utricles shapes at the margins of the graph display the extreme shapes of the morphospace for a better visualization of the utricle morphological features with greater weight within the principal components.

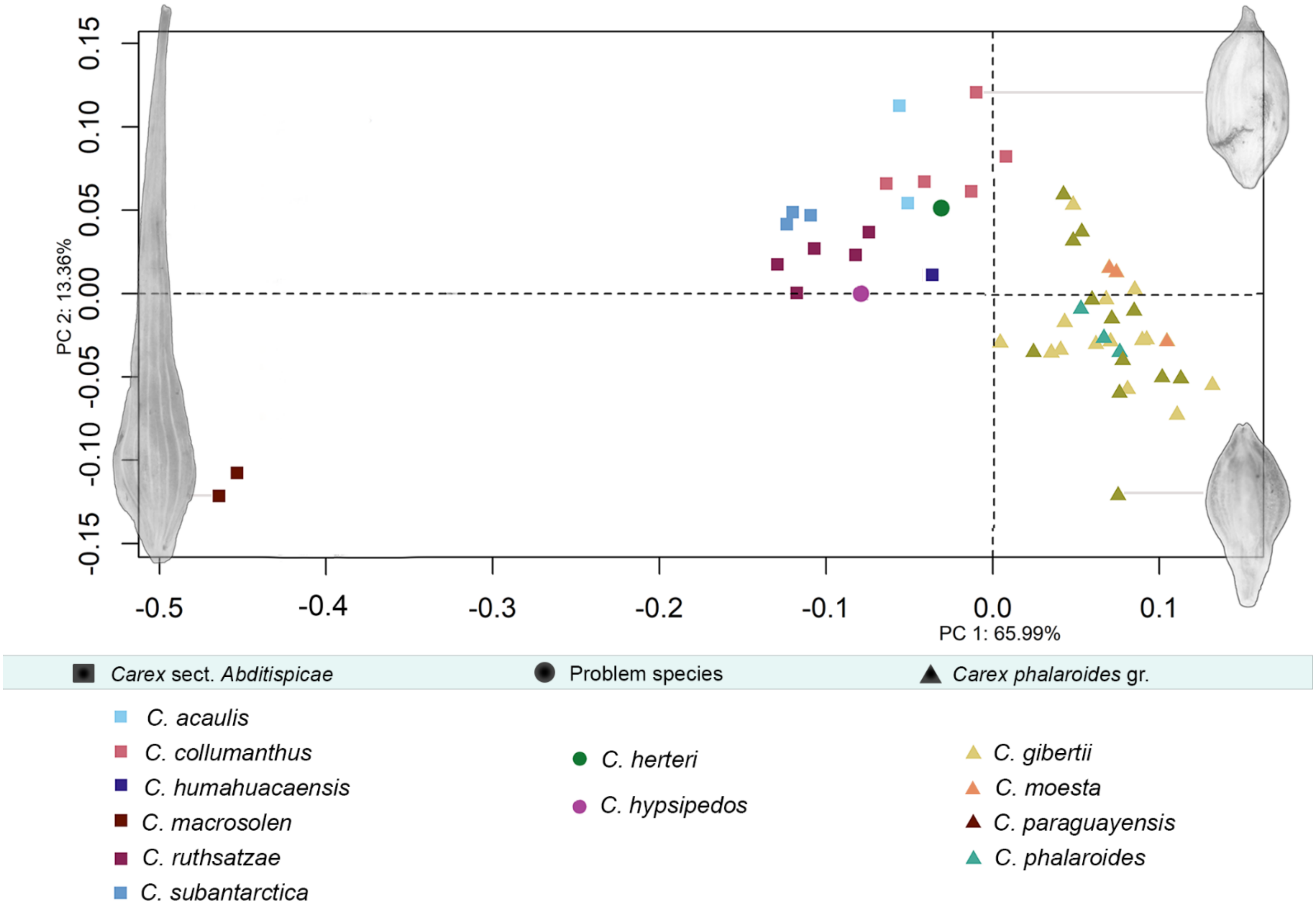

Figure S3. Comparative figure of consensus configurations between datasets: A) Figure representing the utricle consensus configuration (black dots and grey line) and deviations from it (orange dots) for the complete dataset. B) Figure representing the utricle consensus configuration (black dots and blue line) and deviations from it (blue dots) for the sect. *Abditispicae* dataset. C) Figure representing the utricle consensus configuration (black dots and green line) and deviations from it (green dots) for the *C. phalaroides* gr. dataset.

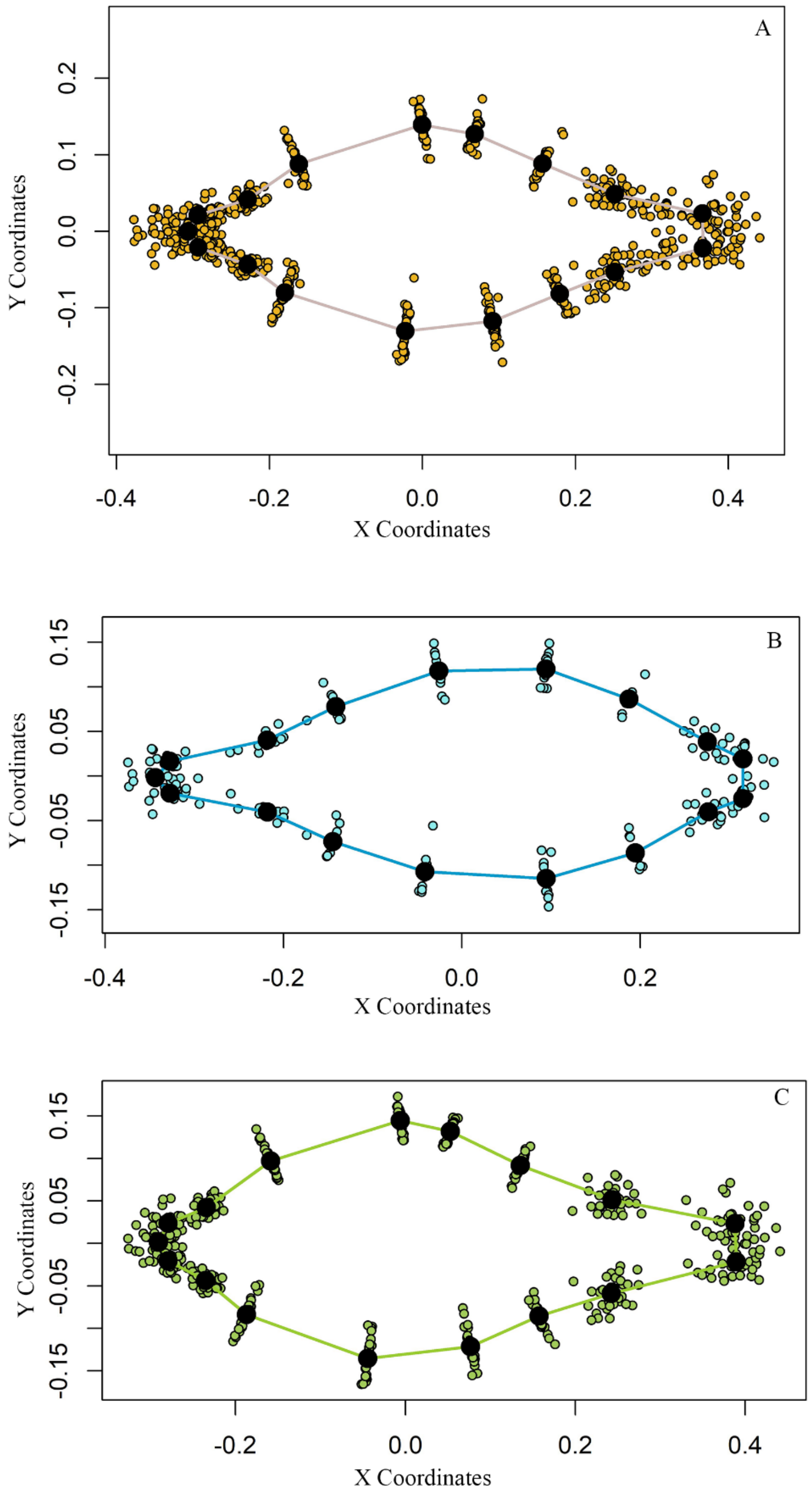

Figure S4. Comparative figure for the consensus utricle configuration of *C. phalaroides* gr. (green triangles) with sect. *Abditispicae* (blue dots).

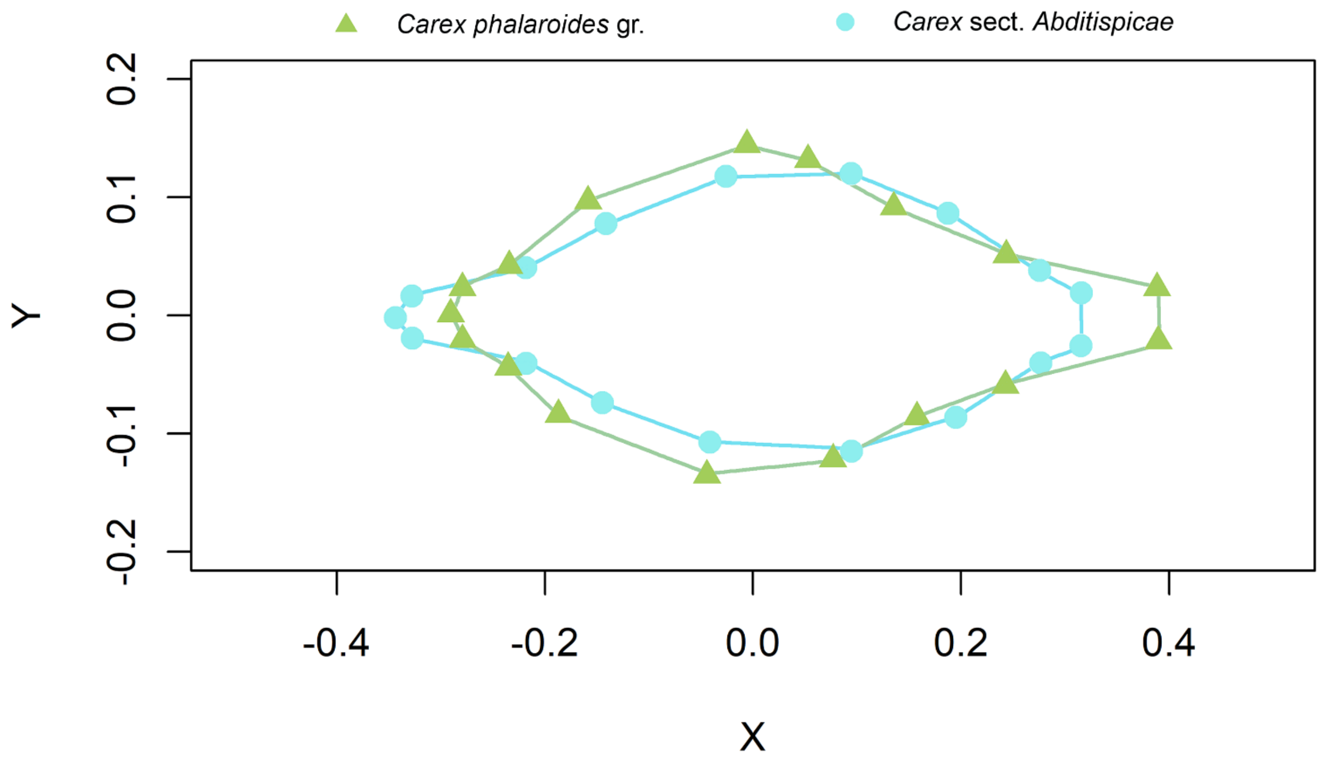

Supplement: Supplementary material 2 — Utricle slide with landmarks; PCA plot with C.macrosolen; Consensus configurations; Group comparison [file phytokeys-232-167_article-100410__-s002.pdf]
